# Safety evaluation of vitamin K2 (menaquinone-7) via toxicological tests

**DOI:** 10.1038/s41598-024-56151-w

**Published:** 2024-03-05

**Authors:** Sung-bong Hwang, Min-ju Choi, Hyeon-jeong Lee, Jeong-jun Han

**Affiliations:** 1 Department of Quality, GF Fermentech, Inc., Sejong, 30077 South Korea; 2 GF Fermentech, Inc., R&D Institute, Daejon, South Korea

**Keywords:** Biological techniques, Biotechnology

## Abstract

This study aims to evaluate the safety of MK-7 produced by fermentation process using a *Bacillus subtilis* var. *natto* strain for human ingestion via acute oral toxicity, repeated dose 90-day oral toxicity, 28-day recovery test, and genotoxicity tests. The acute oral toxicity test results indicated that all subjects survived at the dose of 5000 mg/kg with no toxic effects. For the repeated dose 90-day oral toxicity test, MK-7 was administered to rats at 500, 1500, and 4500 mg/kg for 90 d. No abnormal findings were detected in clinical observations or in clinical pathological and histopathological examinations. The no-observed-adverse-effect level(NOAEL) was determined to be 4500 mg/kg/d, the maximum dose tested. For the evaluation of genotoxicity, reverse mutation, chromosomal aberration, and micronucleus tests were performed. In the reversion mutation test, vitamin K2 did not induce reversion in bacterial strains, and no chromosomal abnormality was observed in the chromosomal abnormality test using Chinese hamster lung cells. In the micronucleus test, micronuclei were not induced using ICR mouse bone marrow cells. All the toxicity test results suggest that vitamin K2 produced by fermentation processes using *Bacillus subtilis* var. *natto* induced no toxicological changes under the experimental conditions.

## Introduction

Vitamin K is a fat-soluble vitamin, with "K" derived from the German word "koagulation" (meaning clotting) and its role in various protein functions related to blood coagulation that have been studied for over 90 years^1^. Vitamin K was first discovered in 1930 by Danish scientist Henrik Dam who found that reducing the intake of vitamin K in chickens induced intramuscular hemorrhage in the subcutaneous fat. Thereafter, the isolation, identification, and characterization of the vitamin K structure and its properties as an anti-hemorrhagic agent were further studied. Although it is well known that vitamin K deficiency can contribute to significant bleeding^2^, the role of vitamin K in improving osteoporosis and arterial calcification has begun to be studied only recently^3^.

Vitamin K is divided into K1 (phylloquinone) and K2 (menaquinone), which have the similar functions but different characteristics depending on their bioavailability and bioactivity^1^. Vitamin K2, the main storage form of vitamin K in animals, is subdivided into homologues depending on the length of the isoprenoid residue. Menaquinone is expressed as "MK-n," where M stands for menaquinone, K for vitamin K, and n for the number of isoprenoid residues. Among them, MK-4 and MK-7 are known to prevent age-related bone loss, improve bone strength and genral cardiovascular health^4^. MK-4 is derived from animal sources such as dairy products, and MK-7 is produced by intestinal bacteria. MK-7 is present in several food substances that have long been consumed by humans, such as cabbage, yogurt, natto and cheese. Today, MK-7 is used in nutritional supplements, beverages, milk, and milk powder. Compared to MK-4, MK-7 has a higher bioavailability due toits longer half life. Considering the increase in the use of MK-7 in nutritious foods and supplements, a systematic toxicological study is required for ensuring the safety of MK-7.

In this study, acute oral toxicity (OECD No. 420), repeated oral administration (OECD No. 408), and genotoxicity (OECD No. 471, 473, and 474) tests were performed to evaluate the safety of vitamin K2 (MK-7) produced through fermentation processes. Furthermore, the study aimed to investigate the toxicological effects of in vivo repeated oral exposure to the dosage of 4500 mg/kg, surpassing the previously reported no observed adverse effect level (NOAEL) of 2000 mg/kg, as reported in existing MK-7 toxicity studies^2,3^.

## Results

### Acute oral toxicity test

In the preliminary study and acute oral administration test, no animals died in any test dose, and no toxic reaction to the test substance was observed in the either study group. No abnormal changes in body weight were observed in either group, and no abnormal findings were observed during autopsy, requiring no statistical treatment. In both studies, all subjects survived at the dose of 5000 mg/kg with no toxic effects. Therefore, the test substance was classified as "category 5" or "unclassified" in the Globally Harmonized System of Classification and Labelling of Chemicals(GHS) classification.

### Repeated dose oral toxicity tests

#### 28-day, repeated (oral) dose determination test

The purpose of this test was to investigate the overall toxic effect on rats after daily oral administration of the test item for 28 days. In the results of food consumption, significant decrease of food consumption was observed in the male vehicle control, female low and high dose group, but it was not considered toxicological significance due to a temporary phenomenon. While the hemoglobin distribution of the male vehicle control group, the low-dose group, and the high-dose group significantly decreased compared to the negative control group, no abnormal changes were observed in other erythrocyte-related indicators. Additionally, the changes were not dose-dependent and no other toxicological changes associated with decreased hemoglobin distribution were identified. In the blood biochemistry results, a decrease in alanine aminotransferase levels was noted in the male groups. However, this change was not considered toxicological as it was observed across all test groups, including vehicle control group. Similarly, a reduction in albumin levels was observed in the male mid-dose group, but it was not deemed toxicologically significant as no dose-dependent trend was evident. Regarding organ weight findings, an increase in the absolute weight of the liver was observed in the male mid-dose group. However, this change was not considered toxicologically significant due to the absence of alterations in relative organ weight and the lack of a dose–response relationship. Overall, no abnormal changes were observed during the observation period in comparison to the negative control group, including general symptom observation, body weight, feed intake, hematological tests, blood biochemical tests, organ weights, and autopsy findings. Based on these results, it was considered appropriate to set the high dose of the 90-d repeated toxicity test as 4500 mg/kg or less.

#### 90-day, repeated dose (oral) toxicological test with 28-day recovery group

No statistically significant changes were observed in weight measurement, water consumption, ophthalmological examination, sperm examination, or vaginal smear examination (Table 1, body weight). Salivation was sporadically observed in the vehicle control group and all test substance administration groups. In the vehicle control group, the low-dose group, and the medium-dose group, dirtiness around the nose, in the lower abdomen, soft stool, mucous stool, and diarrhea were observed sporadically. Feed gnawing was observed in the female medium- and high-dose groups. Salivation, lower abdominal contamination, soft stool, mucous stool, and diarrhea observed during the administration period were likely caused by vehicle administration as the same abnormal changes were observed also in the vehicle control group or only in the vehicle control group. In addition, although feed gnawing was observed in the female medium- and the high-dose groups, it was not considered a toxicologically notable change because it was transiently observed. The feed consumption from week 1 to week 13 of administration decreased sporadically in the vehicle control group and in all test substance administration groups. However feed consumption increased as the concentration of the test substance decreased. Since a decrease in feed consumption was also observed in the vehicle control group, the decrease in feed consumption observed in the main study was considered to have been caused by the vehicle.

**Table 1:** Weight change between repeated dose 90-d oral dose determination tests.

Group/weeks															
Dose (mg/kg bw/day)		0	1	2	3	4	5	6	7	8	9	10	11	12	13
Sex: male (g)															
G1^1^	Mean	198.10	263.87	319.17	366.50	405.41	434.22	459.91	484.55	506.57	523.46	538.29	549.28	559.63	568.34
0	S.D	8.47	13.30	16.24	18.59	24.95	27.08	32.45	34.89	35.79	38.37	40.46	43.38	46.05	45.68
	N	15	15	15	15	15	15	15	15	15	15	15	15	15	15
G2^2^	Mean	196.73	261.90	316.09	360.03	395.58	426.40	452.4	475.14	495.03	512.82	527.06	541.11	550.20	557.60
0	S.D	6.79	13.06	18.32	26.03	32.26	38.18	42.11	45.71	49.85	51.12	53.24	56.34	56.12	57.95
	N	15	15	15	15	15	15	15	15	15	15	15	15	15	15
G3	Mean	194.84	259.99	313.73	359.33	393.63	422.82	450.44	471.03	489.38	506.41	520.67	534.71	543.42	548.21
500	S.D	7.23	9.51	13.24	17.72	19.76	20.72	22.01	23.88	23.40	26.07	28.03	27.41	29.93	30.97
	N	10	10	10	10	10	10	10	10	10	10	10	10	10	10
G4	Mean	197.35	260.35	317.34	366.27	402.79	435.83	463.93	487.01	506.55	525.47	540.84	553.20	561.14	569.69
1500	S.D	8.02	12.64	18.69	24.18	28.98	32.98	36.52	40.61	40.67	46.74	46.86	45.68	49.76	50.85
	N	10	10	10	10	10	10	10	10	10	10	10	10	10	10
G5	Mean	196.48	264.37	320.88	369.57	409.42	440.50	468.8	468.83	514.50	532.36	548.43	562.16	572.46	580.44
4500	S.D	7.37	12.41	19.33	23.50	29.88	35.77	41.1	41.1	46.95	49.33	51.19	51.66	53.59	54.91
	N	15	15	15	15	15	15	15	15	15	15	15	15	15	15
Sex: female															(g)
G1^1^	Mean	147.78	183.17	210.57	235.57	255.14	271.86	281.37	294.69	305.99	312.79	317.45	326.52	331.52	336.04
0	S.D	7.42	7.29	11.80	15.51	17.76	17.05	19.27	19.68	22.70	22.19	24.17	25.86	28.83	26.38
	N	15	15	15	15	15	15	15	15	15	15	15	15	15	15
G2^2^	Mean	148.85	181.67	210.15	232.88	252.88	267.70	278.54	289.99	303.45	307.03	313.72	320.40	325.20	328.74
0	S.D	6.89	7.22	9.91	14.11	12.66	15.26	17.59	19.72	18.02	22.59	24.36	24.40	24.59	24.97
	N	15	15	15	15	15	15	15	15	15	15	15	15	15	15
G3	Mean	151.36	183.33	209.14	226.75	249.69	264.49	275.63	281.92	294.83	301.35	306.28	308.34	316.52	321.01
500	S.D	7.51	7.75	10.62	16.46	13.06	16.01	18.04	19.60	17.75	18.19	19.81	21.76	18.07	18.20
	N	10	10	10	10	10	10	10	10	10	10	10	10	10	10
G4	Mean	150.28	186.37	210.69	232.29	250.50	264.88	278.54	288.92	305.34	308.62	316.09	322.69	323.32	327.06
1500	S.D	7.68	11.54	16.23	15.86	19.11	21.17	22.97	22.68	24.07	25.65	24.45	28.07	30.86	32.82
	N	10	10	10	10	10	10	10	10	10	10	10	10	10	10
G5	Mean	149.61	182.17	208.37	229.78	252.57	265.30	275.53	286.76	302.37	306.21	312.52	322.33	321.74	325.55
4500	S.D	7.38	12.73	17.05	19.75	23.67	26.84	25.50	25.16	28.96	30.85	29.35	33.40	33.09	31.71
	N	15	15	15	15	15	15	15	15	15	15	15	15	15	15

*bw* body weight, *N* animal number, *S.D*. standard deviation.

^1^Negative control, ^2^vehicle control.

In the functional tests, a statistically significant increase in grip strength was observed in the male low-dose group of the main group compared to the negative control group, but no dose-dependent change was observed, indicating no toxicological significance. Urinalysis results showed that epithelial cells were sporadically observed in some subjects of the 90-d group as well as in male and female test substance groups of the recovery group. However, abnormal changes were also observed sporadically in the control groups. All changes were within the range of biological variability or were incidentally distributed (Table 2, Urinalysis results). In the hematological results, the reticulocyte count decreased in the male vehicle control in the recovery group and the prothrombin time increased in the high-dose female recovery group, but no abnormal changes were observed in the main group, indicating no toxicological significance (Table 3, blood test results). As no abnormal changes in the urinary system were observed in the histopathological examination or blood biochemical examination results, all changes were determined to have no toxicological significance.

**Table 2:** Urinalysis results.

Group/dose (mg/kg/bw/day)		Male					Female				
		G1^1^	G2^2^	G3	G4	G5	G1^1^	G2^2^	G3	G4	G5
		0	0	500	1500	4500	0	0	500	1500	4500
No. of animals		5	5	5	5	5	5	5	5	5	5
Volume (mL)	Mean	10.5	9	10	10.3	10	10.6	9.5	9.5	10	10
	S.D	1.3	1.1	0.6	0.6	0.9	1.1	0.9	1.2	0.7	0.6
Color	Paleyellow	2			1		3				
	Yellow	3	5	5	4	5	2	5	5	5	5
Transparency	Clear	5	4	5	5	5	5	5	5	5	5
	Slight turbid		1								
	Turbid										
Glucose	–	5	5	5	5	5	5	5	5	5	5
	Trace(+ –)										
Bilirubin	–	5	5	5	5	5	5	5	5	5	5
	1 +										
Ketone body	–	5	1		3	1	5	4	2	3	4
	1 +		3	3	2	3		1	3	2	1
	2 +		1	2		1					
	3 +										
Specific gravity	1.000										
	1.005						1				
	1.010	3					2				
	1.015	1	1							1	
	1.02	1									
	1.025				1		1		1	1	1
	1.03		4	5	4	5	1	5	4	3	4
Occult blood	–	3	2	3	4	3	5	5	5	5	5
	Trace(+ –)	2	2	2	1	2					
	1 +		1								
	2 +										
	3 +										
pH	5.0							1	1		
	5.5			2	1	1		4	2	3	3
	6		2	2	2	2			1	1	1
	6.5		1	1	2	1					
	7		1								
	7.5		1				2		1	1	1
	8	1				1	1				
	8.5	4									
	9						2				
Protein	–						2				
	Trace(+ –)	2					2				
	1 +	3	1		1	1	1	1	1	2	2
	2 +		2	1	2	2		4	3	2	2
	3 +		2	3	2	1			1	1	1
	4 +			1		1					
Urobilinogen	Normal	5	2	4	5	4	5	5	4	5	5
	1 +		3	1		1			1		
NIT	–	5	4	5	5	4	5	4	4	5	5
	+		1			1		1	1		
Leukocyte	–	4					4	2	1	3	
	1 +	1	3	2	3	3	1	3	3	2	5
	2 +		2	3	2	2			1		
	3 +										
Cast^†^	–	5	5	5	5	5	5	5	5	5	5
	±										
	+										
	+ +										
	+ + +										
Epithelial cell^†^	–	5	5	5	5	5	5	4	5	5	4
	±							1			
	+										
	+ +										
	+ + +										1
Leukocyte^†^	–	5	5	5	5	5	5	5	5	5	5
	±										
	+										
	+ +										
	+ + +										
Erythrocyte^†^	–	5	5	5	5	5	5	5	5	5	5
	±										
	+										
	+ +										
	+ + +										

*BIL* bilirubin, *BLO* occult blood, *CLA* clarity, *COL* color, *GLU* glucose, *KET* ketone body, *LEU* urinary sediment, and leukocyte, *NIT* nitrite, *PRO* protein, *S.D*. standard deviation, *SG* pH specific gravity, *URO* urobilinogen, *VOL* volume.

^†^Sediment, ^1^negative control, ^2^vehicle control.

**Table 3:** Repeated dose 90-day oral toxicity test: blood test results.

Group/dose (mg/kg bw/day)		G1^1^	0		G2^2^	0		G3	500		G4	1500		G5	4500	
		Mean	S.D	N	Mean	S.D	N	Mean	S.D	N	Mean	S.D	N	Mean	S.D	N
Sex: male																
WBC	× 10^3^/μL	9.23	2.53	10	10	2.62	10	8.61	2.62	10	10.76	2.01	10	11.3	2.39	10
RBC	× 10^6^/μL	8.46	0.24	10	8.6	0.5	10	8.34	0.24	10	8.41	0.45	10	8.58	0.32	10
HGB	g/dL	15.5	0.5	10	15.4	0.8	10	15.1	0.6	10	15.2	0.7	10	15.6	0.6	10
HCT	%	44.5	1.1	10	44.4	1.9	10	43.2	1.3	10	43.8	2	10	44.8	1.2	10
MCV	fL	52.7	1.4	10	51.7	1.6	10	51.8	1	10	52.1	1.3	10	52.3	1.9	10
MCH	pg	18.4	0.5	10	18	0.6	10	18.1	0.4	10	18.2	0.6	10	18.2	0.8	10
MCHC	g/dL	34.9	0.5	10	34.7	0.4	10	34.9	0.5	10	34.8	0.6	10	34.9	0.6	10
PLT	× 10^3^/μL	1,073	102	10	1110	89	10	1050	118	10	1087	100	10	1006	147	10
RDW	%	15.8	0.8	10	15.5	1.3	10	15.5	0.9	10	15.1	1.4	10	15.5	1.2	10
MPV	fL	7.8	0.2	10	7.6	0.3	10	7.7	0.2	10	7.6	0.2	10	7.8	0.4	10
NEU	%	20.4	5.7	10	19.1	6.3	10	19	2.8	10	18.1	8.2	10	14.1	3	10
LYM	%	70.9	7.6	10	72.1	7.5	10	71.8	2.8	10	73.1	8.3	10	76	4.3	10
MONO	%	7.2	2	10	7.5	1.6	10	8	1.9	10	7.6	1.6	10	8.7	2.1	10
EOS	%	1.1	0.5	10	1.1	0.3	10	0.9	0.5	10	0.9	0.4	10	0.9	0.2	10
BASO	%	0.4	0.1	10	0.4	0.1	10	0.4	0.1	10	0.3	0.1	10	0.4	0.1	10
Retic	%	3.03	0.53	10	2.55	0.56	10	2.44	0.23	10	2.51	0.36	10	2.51	0.32	10
PT	sec	8.3	0.3	10	8.6	0.4	10	8.5	0.3	10	8.6	0.2	10	8.6	0.3	10
APTT	sec	14.8	0.8	10	14.1	0.8	10	13.9	1.3	10	14.1	1.2	10	13.3	1.2	10
FMetHb	%	0	0	10	0	0	10	0	0.1	10	0.1	0.2	10	0	0	10
HZB	%	0.2	0.2	10	0.2	0.3	10	0.2	0.3	10	0.2	0.4	10	0.1	0.2	10
Sex: female																
WBC	× 10^3^/μL	4.77	1.09	10	4.99	1.54	10	4.87	0.96	10	3.77	1.17	10	5.25	1.68	10
RBC	× 10^6^/μL	7.87	0.44	10	7.69	0.5	10	7.79	0.34	10	7.74	0.27	10	7.79	0.41	10
HGB	g/dL	14.6	0.8	10	14.5	0.5	10	14.6	0.6	10	14.5	0.3	10	14.3	0.5	10
HCT	%	42.1	2	10	41.6	1.3	10	42	1.3	10	41.7	0.9	10	41.5	2	10
MCV	fL	53.5	1.5	10	54.2	2	10	54	1	10	53.9	1.6	10	53.2	1.4	10
MCH	pg	18.6	0.5	10	18.9	0.8	10	18.8	0.3	10	18.8	0.5	10	18.4	0.5	10
MCHC	g/dL	34.8	0.4	10	34.8	0.3	10	34.8	0.4	10	34.9	0.4	10	34.5	0.5	10
PLT	× 10^3^/μL	1034	136	10	1026	67	10	1055	201	10	1028	94	10	1012	67	10
RDW	%	13	0.7	10	12.2	1.4	10	12.4	0.7	10	12.3	0.4	10	12.5	0.8	10
MPV	fL	7.9	0.2	10	7.7	0.2	10	8	0.5	10	7.9	0.3	10	8	0.2	10
NEU	%	17.4	4.8	10	18.2	6.2	10	20.7	7.5	10	16.2	5.5	10	16.2	4.5	10
LYM	%	73.9	6	10	73.3	7	10	69.4	7.4	10	75.8	5.7	10	75.4	4.8	10
MONO	%	7.2	1.5	10	7.2	2.2	10	8.5	1.7	10	6.5	2.2	10	7.4	1.7	10
EOS	%	1.3	0.6	10	0.9	0.3	10	1	0.3	10	1.3	0.6	10	0.9	0.4	10
BASO	%	0.3	0.1	10	0.4	0.1	10	0.4	0.1	10	0.3	0.2	10	0.2	0.1	10
Retic	%	2.72	0.41	10	2.63	0.48	10	2.81	0.65	10	2.58	0.38	10	2.65	0.5	10
PT	sec	7.5	0.2	10	7.6	0.3	10	7.7	0.3	10	7.6	0.2	10	7.6	0.2	10
APTT	sec	12.7	1.2	10	12	1	10	12.6	1.6	10	12.5	1.2	10	12.1	1.5	10
FMetHb	%	0.5	0.4	10	0.5	0.3	10	0.5	0.4	10	1	0.4	10	0.9	0.4	10
HZB	%	0.3	0.3	10	0.4	0.5	10	0.5	0.5	10	0.6	0.7	10	0.5	0.6	10

*APTT* activated partial thromboplastin time, *BASO* basophil, *bw* body weight, *EOS* eosinophil, *FMetHb* met hemoglobin, *HCT* hematocrit, *HGB* hemoglobin, *HZB* Heinz body, *LYM* lymphocyte, *MCH* mean corpuscular hemoglobin, *MCHC* mean corpuscular hemoglobin concentration, *MCV* mean corpuscular volume, *MONO* monocyte, *MPV* mean platelet volume, *N* animal number, *NEU* WBC differential counting, neutrophil, *PLT* platelet, *PT* prothrombin time, *RBC* total erythrocyte count, *RDW* RBC distribution width, *Retic* reticulocyte, *S.D*. standard deviation and *WBC*: total leukocyte count.

^1^negative control, ^2^vehicle control.

In the blood biochemical analysis, total cholesterol significantly decreased in the main group males, both in the low-dose and high-dose groups, compared to the negative control group. In the medium-dose group, total cholesterol, sodium, and chloride exhibited statistically significant reductions compared to the negative control group. In the high-dose group, triglycerides showed a statistically significant increase compared to the negative control group, while sodium and chloride exhibited statistically significant decreases. In the main group females, both the low-dose and high-dose groups showed a statistically significant decrease in urea compared to the negative control group. These changes were considered to have no toxicological significance, as dose-dependent changes were not observed. In the male excipient control group of the recovery group, albumin level and albumin/globulin (A/G) ratio) were statistically significantly increased compared with the negative control group. In the female high-dose group of the recovery group, the A/G ratio was statistically significantly reduced compared with the negative control group. In the male mid-dose group of the main group, thyroxine hormone level was statistically significantly reduced compared with the negative control group. In the female group of the main group and the test-article administration group of the recovery group, no statistically significant changes were observed compared with the negative control group. A statistically significant increase in the relative organ weight of the lung was observed in the male high-dose group of the recovery group compared with the negative control group (Tables 4, 5). In the autopsy findings, one case of yellow spots on the skin of the left epididymis was observed in the male high-dose group of the main study. The focal monocytic cell infiltration in the Harderian gland, the focal monocytic cell infiltration in the heart, the polysomal basophilic tubule in the kidney, the vitreous column; monocytic cell infiltration and mineralization in the interstitium, polymorphic monocytic cell infiltration in the liver, multifocal necrosis and sporadic hepatocellular vacuolization, cysts in the middle of the pituitary gland, gill microsomal sacs in the thyroid gland, and mononuclear cell infiltration in the interstitium of the prostate gland observed in both the main and recovery groups are lesions commonly observed in SD rats of the same week of age and were distributed incidentally or sporadically, indicating no toxicological significance.

**Table 4:** Repeated dose 90-day oral toxicity test: hematological test results.

Group/dose (mg/kg bw/day)		G1^1^	0		G2^2^	0		G3	500		G4	1500		G5	4500	
		Mean	S.D	N	Mean	S.D	N	Mean	S.D	N	Mean	S.D	N	Mean	S.D	N
Sex: male																
AST	U/L	139	77	10	187	182	10	113	22	10	184	195	10	131	71	10
ALT	U/L	51.9	55.5	10	47.9	44.7	10	26.4	4.3	10	56.1	78.2	10	37.7	31.7	10
ALP	U/L	312	60	10	249	49	10	243	48	10	276	63	10	305	64	10
BUN	mg/dL	15.2	1.7	10	11.9	1.9	10	12.9	2	10	13.5	5	10	13	1.4	10
CRE	mg/dL	0.51	0.05	10	0.48	0.04	10	0.49	0.04	10	0.46	0.04	10	0.48	0.03	10
T-BIL	mg/dL	0.03	0.02	10	0.03	0.03	10	0.02	0.01	10	0.02	0.02	10	0.02	0.02	10
CK	U/L	956	413	10	760	338	10	963	432	10	923	272	10	970	240	10
GLU	mg/dL	131	12	10	126	11	10	129	15	10	130	14	10	136	16	10
T-CHO	mg/dL	84	17	10	63*	8	10	65*	12	10	65*	15	10	66	10	10
TG	mg/dL	45	21	10	80	34	10	74	25	10	64	20	10	97*	50	10
IP	mg/dL	6.6	0.6	10	6.6	0.6	10	6.7	0.7	10	6.7	0.7	10	6.3	0.7	10
Ca	mg/dL	10.3	0.3	10	10.4	0.2	10	10.1	0.3	10	10.1	0.3	10	10.3	0.4	10
TP	g/dL	6.4	0.2	10	6.2	0.3	10	6	0.3	10	6	0.4	10	6.1	0.3	10
ALB	g/dL	2.5	0.1	10	2.5	0.1	10	2.5	0.1	10	2.4	0.1	10	2.4	0.1	10
HDL	mg/dL	23.9	2.7	10	22.9	2.4	10	22.1	3.1	10	22.6	3.4	10	22.5	1.9	10
LDL	mg/dL	6.8	2.4	10	5.3	0.9	10	6.7	2.4	10	6.3	1.5	10	6.1	1.7	10
CHE	U/L	166	68	10	241	318	10	160	53	10	138	34	10	144	32	10
TBA	umol/L	24.4	20.8	10	38.1	39.7	10	24.9	14.4	10	32.2	39.4	10	35.5	35.2	10
UREA	mmol/L	33	5	10	26	5	10	29	6	10	30	10	10	30	4	10
A/G	ratio	0.66	0.04	10	0.68	0.06	10	0.7	0.06	10	0.69	0.05	10	0.67	0.04	10
Na	mmol/L	142	2	10	140	1	10	140	1	10	140*	1	10	140*	1	10
K	mmol/L	4.8	0.2	10	4.9	0.2	10	4.8	0.2	10	5	0.2	10	5	0.2	10
Cl	mmol/L	106	2	10	105	1	10	105	1	10	104*	1	10	104*	1	10
Sex: female																
AST	U/L	116	39	10	96	11	10	99	19	10	97	22	10	122	36	10
ALT	U/L	39.3	26.6	10	24.3	10.7	10	27.3	15.1	10	22	9.3	10	26.3	15.9	10
ALP	U/L	147	64	10	152	71	10	138	56	10	153	69	10	169	69	10
BUN	mg/dL	14.9	1.3	10	13.8	3.1	10	12.3	1.2	10	13.1	1.6	10	14.3	3	10
CRE	mg/dL	0.48	0.08	10	0.52	0.1	10	0.49	0.04	10	0.49	0.06	10	0.5	0.05	10
T-BIL	mg/dL	0.04	0.02	10	0.05	0.03	10	0.03	0.02	10	0.04	0.02	10	0.06	0.05	10
CK	U/L	1236	342	10	1636	853	10	1415	666	10	1624	570	10	1272	566	10
GLU	mg/dL	135	20	10	141	11	10	123	15	10	127	12	10	140	18	10
T-CHO	mg/dL	79	12	10	74	18	10	81	26	10	89	17	10	85	21	10
TG	mg/dL	18	8	10	31	15	10	29	10	10	30	20	10	25	13	10
IP	mg/dL	6	0.7	10	5.9	0.6	10	5.9	0.7	10	5.6	0.5	10	5.9	0.7	10
Ca	mg/dL	9.8	0.6	10	9.9	0.4	10	10	0.4	10	9.9	0.1	10	9.5	1.1	10
TP	g/dL	6.5	0.5	10	6.4	0.4	10	6.6	0.6	10	6.6	0.2	10	6.6	0.4	10
ALB	g/dL	2.9	0.3	10	2.9	0.2	10	3	0.4	10	2.9	0.1	10	2.9	0.3	10
HDL	mg/dL	25.4	2.3	10	25.1	4.5	10	26.6	6.1	10	28.8	3.8	10	26.7	3.6	10
LDL	mg/dL	4	1.1	10	3.3	0.7	10	3.9	1	10	4.5	1.1	10	4.1	0.8	10
CHE	U/L	1441	399	10	1,297	283	10	1263	336	10	1251	333	10	1208	422	10
TBA	umol/L	23	14.3	10	22.2	19.2	10	27.7	20.6	10	20.3	16	10	23	14.3	10
UREA	mmol/L	34	3	10	29	6	10	26*	2	10	29	3	10	25*	4	10
A/G	ratio	0.78	0.08	10	0.81	0.08	10	0.81	0.07	10	0.8	0.05	10	0.78	0.08	10
Na	mmol/L	141	1	10	141	1	10	141	2	10	141	1	10	140	1	10
K	mmol/L	4.7	0.3	10	4.7	0.2	10	4.7	0.3	10	4.6	0.5	10	4.7	0.3	10
Cl	mmol/L	106	2	10	106	1	10	105	1	10	106	1	10	106	2	10

*A/G*:albumin/globulin ratio, *ALB* albumin, *ALP* alkaline phosphatase, *ALT* alanine aminotransferase, *AST* aspartate aminotransferase, *BUN* blood urea nitrogen, *bw* body weight, *Ca* calcium ion, *CHE* cholinesterase, *CHO* total cholesterol, *CK* creatine kinase, Cl* chloride, *CRE* creatinine, *GLU* glucose, *HDL* high density lipoprotein cholesterol, *IP* inorganic phosphorus, K* Potassium, *LDL* low-density lipoprotein cholesterol, *N* animal number, Na* sodium, *S.D*. standard deviation, *TBA* total bile acid, *T*-*BIL* total bilirubin, *TG* triglyceride, *T-TP* total protein and *UREA* urea. *Measured with an electrolyte analyzer.

^1^Negative control, ^2^vehicle control. *Significantly different from G1 by ANOVA-test, p < 0.05.

**Table 5:** Repeated dose 90-d oral toxicity test: absolute organ weight.

Group/dose (mg/kg bw/day)	G1^1)^	0		G2^2)^	0		G3	500		G4	1500		G5	4500	
	Mean	S.D	N	Mean	S.D	N	Mean	S.D	N	Mean	S.D	N	Mean	S.D	N
Sex: male															
B.W	540.33	45.85	10	533.93	58.32	10	526.95	29.44	10	546.78	48.22	10	563.00	51.19	10
Pituitary gland	0.0133	0.0014	10	0.0119	0.0020	10	0.0121	0.0021	10	0.0118	0.0019	10	0.0124	0.0021	10
Adrenal glands	0.0623	0.0058	10	0.0550	0.0184	10	0.0632	0.0135	10	0.0618	0.0117	10	0.0571	0.0081	10
Thymus	0.3137	0.0352	10	0.3024	0.0617	10	0.2856	0.0802	10	0.3304	0.0759	10	0.3603	0.0758	10
Prostate	3.2398	0.2599	10	3.2406	0.5560	10	3.1693	0.4384	10	2.8362	0.5395	10	3.1924	0.3994	10
Spleen	0.8288	0.1140	10	0.7836	0.1214	10	0.7905	0.1133	10	0.7789	0.0829	10	0.8317	0.1013	10
Lung	1.5701	0.1395	10	1.6157	0.1613	10	1.5943	00.1761	10	1.6433	0.1137	10	1.6735	0.1938	10
Heart	1.4702	0.1698	10	1.4680	0.1912	10	1.4412	0.0895	10	1.4583	0.1337	10	1.5180	0.1431	10
Brain	2.1737	0.0873	10	2.0746	0.0839	10	2.1617	0.1129	10	2.1714	0.1078	10	2.1643	0.1189	10
Epididy mides	1.6157	0.1175	10	1.6113	0.1041	10	1.6398	0.1512	10	1.5729	0.0919	10	1.6319	0.1163	10
Testis	3.5053	0.2926	10	3.5104	0.2945	10	3.4361	0.3049	10	3.5624	0.2014	10	3.4618	0.2458	10
Kidneys	3.0375	0.3207	10	3.1220	0.3977	10	2.9820	0.2754	10	3.1843	0.4436	10	3.3010	0.2722	10
Liver	13.3398	1.8767	10	14.2400	2.0940	10	12.9149	1.0929	10	13.5522	2.3282	10	15.2398	2.4124	10
Thyroids gland	0.0265	0.0055	10	0.0258	0.0033	10	0.0252	0.0081	10	0.0260	0.0039	10	0.0266	0.0035	10
Sex: female (g)															
Body weight	315.41	26.42	10	317.65	24.16	10	304.39	18.90	10	311.09	30.52	10	307.76	32.01	10
Pituitary gland	0.0185	0.0043	10	0.0187	0.0028	10	0.0197	0.0039	10	0.0193	0.0034	10	0.0198	0.0037	10
Adrenal glands	0.0717	0.0079	10	0.0728	0.0066	10	0.0773	0.0092	10	0.0731	0.0140	10	0.0777	0.0080	10
Ovary	0.0993	0.0171	10	0.0915	0.0190	10	0.0778	0.0249	10	0.0830	0.0184	10	0.0887	0.0285	10
Uterus	0.7096	0.2908	10	0.7716	0.1568	10	0.6596	0.1365	10	0.7380	0.2129	10	0.7052	0.2043	10
Thymus	0.3387	0.0523	10	0.2992	0.0584	10	0.2757	0.0581	10	0.3104	0.1112	10	0.3135	0.0875	10
Spleen	0.5618	0.0616	10	0.5659	0.0787	10	0.5464	0.0477	10	0.5424	0.0894	10	0.5427	0.0756	10
Lung	1.3162	0.0956	10	1.3377	0.0935	10	1.2900	0.0962	10	1.3011	0.1307	10	1.2950	0.1020	10
Heart	1.0230	0.1020	10	1.0744	0.0799	10	1.0691	0.0872	10	1.0528	0.0787	10	1.1003	0.0894	10
Brain	2.0453	0.1012	10	2.0121	0.0514	10	1.9678	0.0701	10	2.0183	0.1009	10	2.0005	0.0543	10
Kidneys	1.9396	0.2057	10	1.9300	0.1264	10	1.9232	0.1340	10	1.8965	0.1347	10	2.0343	0.2944	10
Liver	7.6848	0.9612	10	8.4238	0.7512	10	8.3561	0.9547	10	8.5156	0.8149	10	8.5469	1.0836	10
Thyroids gland	0.0277	0.0051	10	0.0315	0.0057	10	0.0269	0.0037	10	0.0306	0.0062	10	0.0310	0.0055	10

^1^negative control, ^2^vehicle control.

Overall, the various examined parameters, including the general and detailed symptom observation, weight measurement, feed and water consumption measurement, functional tests, ophthalmologic examination, urinalysis, blood biochemical and hematological examination, hormone examination, sperm examination, vaginal smear, absolute and relative organ weight measurement, visual inspection during autopsy, and histopathological examination, showed no toxicologically significant changes caused by the test substance. Therefore, the NOAEL was determined to be 4500 mg/kg/d for both females and males.

### Genotoxicity tests

#### Bacterial reverse mutation test

**Figure 1:**
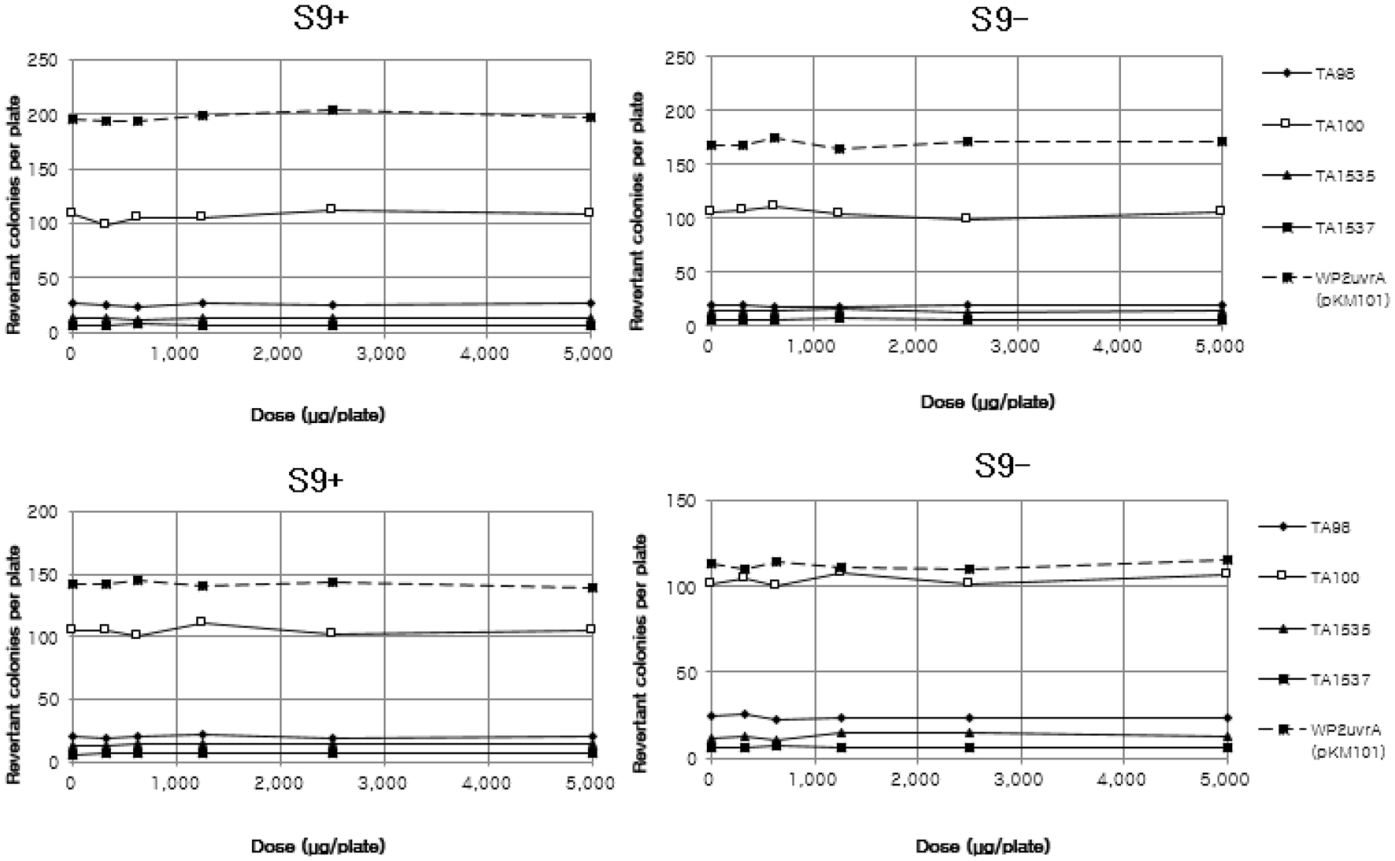
Dose–response curve of revertant colony numbers of differential bacterial strains. S9 + :The presence of metabolic activation system, S9–: The absence of metabolic activation system. Experiments were conducted in duplicate. The reverse mutation of the test substance was evaluated using histidine-requiring test strains (*Salmonella* *typhimurium* TA98, TA100, TA1535, TA1537) and tryptophan-requiring test strains (*E*. *coli* WP2 uvrA/pKM101).

In the bacterial reverse mutation test, which utilized histidine-requiring bacterial test strains (*Salmonella typhimurium* TA98, TA100, TA1535, TA1537) and tryptophan-requiring bacterial test strain (*E. coli* WP2 uvrA/pKM101), no precipitation or inhibition of growth induced by the test substance was observed at any doses in all the strains with or without a metabolic activation system(S9). Therefore, the doses of all the strains in the presence and absence of a metabolic activation system were set in 5 levels at a dose progression factor of 2, with a maximum dose of 5000 µg/plate in the main study. No dose-dependent increase in reverted colonies induced by the test substance was observed at any dose in any strain with or without a metabolic activation system compared to the negative control group. The number of reverted colonies was recorded, and the mean and standard deviation were calculated, followed by no statistical tests (Fig. 1). The number of reverted mutant colonies in the negative control group was within the historical data range, and the number of reverted colonies in the positive control group was significantly increased compared to the negative control group, indicating a clear positive reactionand validating the test. Based on these results, the test substance did not induce reverse mutation in the bacterial strains (Tables 6, 7).

**Table 6:** Number of revertant colonies per plate in the presence and absence of metabolic activation system (1st).

Strain	Dose (μg/plate)	Number of revertant colonies/plate presence of metabolic activation				Dose (μg/plate)	Number of revertant colonies /plate absence of metabolic activation			
		Colony no			Mean ± S.D		Colony no			Mean ± S.D
TA 98	0	27	25	26	26 ± 1	0	19	20	17	19 ± 2
	312.5	26	25	25	25 ± 1	312.5	20	20	18	19 ± 1
	625	23	23	25	24 ± 1	625	18	17	17	17 ± 1
	1250	28	26	26	27 ± 1	1250	17	19	15	17 ± 2
	2500	24	26	26	25 ± 1	2500	17	21	19	19 ± 2
	5000	26	27	25	26 ± 1	5000	19	18	21	19 ± 2
	2-AA(0.5)	273	254	300	276 ± 23	2-NF(1.0)	244	241	252	246 ± 6
TA 100	0	111	106	106	108 ± 3	0	107	112	98	106 ± 7
	312.5	96	97	104	99 ± 4	312.5	95	112	114	107 ± 10
	625	92	111	110	104 ± 11	625	102	114	116	111 ± 8
	1250	97	108	109	105 ± 7	1250	110	104	98	104 ± 6
	2500	114	107	114	112 ± 4	2500	98	97	100	98 ± 2
	5000	105	116	104	108 ± 7	5000	101	102	114	106 ± 7
	B[a]P(2.0)	688	726	516	643 ± 112	SA(1.0)	504	592	736	611 ± 117
TA 1535	0	11	13	15	13 ± 2	0	11	14	16	14 ± 3
	312.5	14	11	16	14 ± 3	312.5	12	18	13	14 ± 3
	625	10	14	11	12 ± 2	625	14	14	13	14 ± 1
	1250	13	13	15	14 ± 1	1250	16	17	13	15 ± 2
	2500	12	13	15	13 ± 2	2500	13	12	15	13 ± 2
	5000	12	14	14	13 ± 1	5000	16	11	15	14 ± 3
	2-AA(2.0)	229	336	336	300 ± 62	SA(1.0)	720	600	744	688 ± 77
TA 1537	0	6	8	6	7 ± 1	0	5	5	7	6 ± 1
	312.5	8	6	6	7 ± 1	312.5	6	6	6	6 ± 0
	625	8	7	7	7 ± 1	625	5	6	6	6 ± 1
	1250	5	8	8	7 ± 2	1250	6	7	7	7 ± 1
	2500	6	7	8	7 ± 1	2500	5	5	8	6 ± 2
	5000	6	6	7	6 ± 1	5000	6	6	7	6 ± 1
	2-AA(2.0)	181	243	202	209 ± 32	9-AA(80.0)	526	518	522	522 ± 4
W P 2uvrA (pKM101)	0	206	192	190	196 ± 9	0	165	177	162	168 ± 8
	312.5	189	192	198	193 ± 5	312.5	162	172	170	168 ± 5
	625	182	211	190	194 ± 15	625	177	175	170	174 ± 4
	1250	193	198	204	198 ± 6	1250	165	169	161	165 ± 4
	2500	192	209	208	203 ± 10	2500	178	172	162	171 ± 8
	5000	191	183	216	197 ± 17	5000	169	171	174	171 ± 3
	2-AA(20.0)	2448	1972	1760	2060 ± 352	AF-2(0.005)	1176	1056	1080	1104 ± 63

*2-AA* 2-aminoanthracene, 9-*AA* : 9-aminoacridine, *AF-2* : 2-(2-furyl)-3-(5-nitro-2-furyl) acrylamide), *B[a]P* benzo[a]pyrene, 2-*NF* 2-nitrofluorene, *SA* sodium azide, *S.D*. standard deviation.

**Table 7:** Number of revertant colonies per plate in the presence and absence of metabolic activation system (2nd).

Strain	Dose (μg/plate)	Number of revertant colonies/plate presence of metabolic activation				Dose (μg/plate)	Number of revertant colonies/plate absence of metabolic activation			
		Colony no			Mean ± S.D		Colony no			Mean ± S.D
TA 98	0	18	20	21	20 ± 2	0	24	23	26	24 ± 2
	312.5	19	19	20	19 ± 1	312.5	25	26	27	26 ± 1
	625	20	17	22	20 ± 3	625	22	21	24	22 ± 2
	1250	18	23	22	21 ± 3	1250	26	23	23	24 ± 2
	2500	20	20	18	19 ± 1	2500	23	23	25	24 ± 1
	5000	20	20	22	21 ± 1	5000	26	22	23	24 ± 2
	2-AA(0.5)	139	131	133	134 ± 4	2-NF(1.0)	323	316	289	309 ± 18
TA 100	0	115	98	103	105 ± 9	0	108	97	97	101 ± 6
	312.5	122	103	90	105 ± 16	312.5	102	108	104	105 ± 3
	625	96	98	108	101 ± 6	625	93	106	102	100 ± 7
	1250	109	110	115	111 ± 3	1250	107	112	104	108 ± 4
	2500	115	98	92	102 ± 12	2500	115	90	98	101 ± 13
	5000	100	109	107	105 ± 5	5000	113	102	106	107 ± 6
	B[a]P(2.0)	728	764	672	721 ± 46	SA(1.0)	660	620	504	595 ± 81
TA 1535	0	14	13	13	13 ± 1	0	9	15	12	12 ± 3
	312.5	13	12	13	13 ± 1	312.5	13	14	10	12 ± 2
	625	15	13	13	14 ± 1	625	11	10	10	10 ± 1
	1250	13	15	15	14 ± 1	1250	14	12	18	15 ± 3
	2500	11	16	16	14 ± 3	2500	14	17	12	14 ± 3
	5000	13	15	16	15 ± 2	5000	10	16	13	13 ± 3
	2-AA(2.0)	327	422	317	355 ± 58	SA(1.0)	412	487	422	± 61
TA 1537	0	6	6	5	6 ± 1	0	7	6	7	7 ± 1
	312.5	6	7	7	7 ± 1	312.5	7	5	8	7 ± 2
	625	5	8	8	7 ± 2	625	7	6	8	7 ± 1
	1250	6	6	7	6 ± 1	1250	8	6	6	7 ± 1
	2500	5	7	8	7 ± 2	2500	7	7	5	6 ± 1
	5000	5	7	6	6 ± 1	5000	8	5	6	6 ± 2
	2-AA(2.0)	155	201	228	195 ± 37	9-AA(80.0)	566	611	630	602 ± 33
W P 2uvrA (pKM101)	0	143	134	148	142 ± 7	0	105	116	118	113 ± 7
	312.5	143	144	140	142 ± 2	312.5	101	110	117	109 ± 8
	625	138	151	144	144 ± 7	625	115	115	112	114 ± 2
	1250	136	139	145	140 ± 5	1250	107	109	117	111 ± 5
	2500	146	140	146	144 ± 3	2500	100	113	118	110 ± 9
	5000	133	141	142	139 ± 5	5000	118	114	115	116 ± 2
	2-AA(20.0)	2116	1980	2411	2169 ± 220	AF-2(0.005)	1768	1528	1729	1675 ± 129

*2-AA* 2-aminoanthracene, 9-*AA* 9-aminoacridine, *AF*-2 2-(2-furyl)-3-(5-nitro-2-furyl) acrylamide), *B[a]P* benzo[a]pyrene, 2-*NF* 2-nitrofluorene, *SA* sodium azide, *S.D*. standard deviation.

#### Chromosomal aberration test

In the short-term treatment group with and without a metabolic activation system in the Chinese hamster lung cells, the frequencies of chromosomal structural aberrations (See Table 8 for the types of chromoasomal aberrations monitored for duing this study) in the negative control group, vehicle control group, and groups treated with 62.5, 125, and 250 g/mL of the test substance were 0.0, 0.0, 0.3, 0.0, and 0.3%, respectively. In the continuous treatment group without a metabolic activation system, the frequencies of chromosomal structural aberrations in the negative control group, vehicle control group, and groups treated with 31.3, 62.5, and 125 g/mL of the test substance were 0.0, 0.0, 0.0, 0.0, and 0.0%, respectively. As a result, no dose-dependent increase or statistically significant increase was observed in terms of structural aberrations in the test substance groups of the short-term treatment group and the continuous treatment group with or without a metabolic activation system compared to the vehicle control group, and no statistically significant increase was observed in the vehicle control group compared to the negative control group. Furthermore, the frequency of chromosomal structural aberrations in the positive control group of the short-term treatment group and the continuous treatment group with or without a metabolic activation system was 14.3, 20.7, and 23.7%, indicating a significant difference from the vehicle control group (p < 0.05) and confirming that the test was performed properly. No statistically significant difference was observed in terms of numerical aberrations in the test substance groups and positive control groups with or without a metabolic activation system compared to the vehicle control group, and no statistically significant increase was observed in the vehicle control group compared to the negative control group (Tables 9, 10) (see Supplementary Table S1 online).

**Table 8:** Types of chromosomal aberrations.

Structural aberration	Chromatid gap (ctg)	Chromosome gap (csg)
	Chromatid break (ctb)	Chromosome break (csb)
	Chromatid exchange (cte)	Chromosome exchange (cse)
	Fragment (frg)	
Numerical aberration	Polyploid (poly)	Endoreduplication (endo)

**Table 9:** Chromosomal aberration: main study results.

Test substance	S9 mix	Dose (ug/mL)	No. of cell analyzed	Structural aberrations			Numerical aberrations	
				Gap(–) aberration cell (%)	Gap( +) aberration cell (%)	Total normal cell	Aberration cell (%)	Total normal cell
Water for injection	+ 6 h	0	300	0.0	0.0	300	0.0	300
Acetone		0	300	0.0	0.0	300	0.0	300
MK-7		62.5	300	0.3	0.7	298	0.3	299
		125	300	0.0	0.3	299	0.0	300
		250	300	0.3	1.0	297	0.0	300
B[a]P		20	300	14.3*	19.3	242	0.3	299
Water for injection	– 6 h	0	300	0.0	0.7	298	0.3	299
Acetone		0	300	0.0	0.3	299	0.0	300
MK-7		62.5	300	0.0	0.0	300	0.3	299
		125	300	0.0	0.0	298	0.0	300
		250	300	0.3	0.7	225	0.7	298
MMC		0.1	300	20.7*	25.0	300	0.3	299
Water for injection	– 24 h	0	300	0.0	0.0	300	0.0	300
Acetone		0	300	0.0	0.0	300	0.0	300
MK-7		31.3	300	0.0	0.0	300	0.0	300
		62.5	300	0.0	0.3	299	0.0	300
		125	300	0.0	1.3	296	0.0	300
MMC		0.1	300	23.7*	32.0	204	0.3	299

*B[a]P* benzo[a]pyrene, *MMC* mitomycin C.

*p < 0.05, significant differences between control and treatment group by Fisher's exact test.

**Table 10:** Frequency of structural chromosomal abnormalities in the positive control group of the short-term treatment group and the continuous treatment group with or without a metabolic activation system.

Test article	S9 mix	Dose (ug/mL)	No. of cell analyzed	Poly	Endo	Aberration cell		Total normal cell
						No	(%)	
Water for injection	+ 6 h	0	150	0	0	0	0.0	300
			150	0	0	0		
Acetone		0	150	0	0	0	0.0	300
			150	0	0	0		
MK-7		62.5	150	0	0	0	0.3	299
			150	1	0	1		
		125	150	0	0	0	0.0	300
			150	0	0	0		
		250	150	0	0	0	0.0	300
			150	0	0	0		
B[a]P		20	150	1	0	1	0.3	299
			150	0	0	0		
Water for injection	− 6 h	0	150	1	0	1	0.3	299
			150	0	0	0		
Acetone		0	150	0	0	0	0.0	300
			150	0	0	0		
MK-7		62.5	150	1	0	1	0.3	299
			150	0	0	0		
		125	150	0	0	0	0.0	300
			150	0	0	0		
		250	150	1	0	1	0.7	298
			150	1	0	1		
B[a]P		0.1	150	0	0	0	0.3	299
			150	0	1	1		
Water for injection	– 24 h	0	150	0	0	0	0.0	300
			150	0	0	0		
Acetone		0	150	0	0	0	0.0	300
			150	0	0	0		
MK-7		31.3	150	0	0	0	0.0	300
			150	0	0	0		
		62.5	150	0	0	0	0.0	300
			150	0	0	0		
		125	150	0	0	0	0.0	300
			150	0	0	0		
B[a]P		0.1	150	1	0	1	0.3	299
			150	0	0	0		

*B[a}P* benzo[a]pyrene, *MMC* mitomycin C.

#### In vivo mouse micronucleus test

In the main study, in which the frequency of chromosomal aberrations or mitotic aberrations induced by the test substance in vivo with micronucleus inducibility in ICR mouse bone marrow cells were examined to assess genotoxicity, no general symptoms or deaths were observed in the animals treated, with no morbidity or mortality confirmed. Comparison of the body weight between groups showed no statistically significant differences. There was no significant increase in the frequency of micronucleated polychromatic erythrocytes among all polychromatic erythrocytes in the test substance group compared to the negative control group or the vehicle control group. Additionally, no significant difference was observed in the frequency ratio of polychromatic erythrocytes to total erythrocytes compared to the negative control group in all dose groups (Tables 11, 12 and 13). However, in the positive control group, the frequency of micronucleated polychromatic erythrocytes among all polychromatic erythrocytes was significantly increased compared to that in the negative control group.

**Table 11:** General symptoms and death rates of animals in micronucleus test.

Test substance	Route	Dose (mg/kg)	No. of animals	Mortality (%)	Morbidity (%)	Clinical signs	Days after dosing								
							1		2					3	
							1st	2nd	1st	2nd	1st	3rd	4th	1st	2nd
Water for injection	P.O	0	5	0	0	NAD	5	5	5	5	5	5	5	5	5
MCT Oil	P.O	0	5	0	0	NAD	5	5	5	5	5	5	5	5	5
MK-7	P.O	500	5	0	0	NAD	5	5	5	5	5	5	5	5	5
		1000	5	0	0	NAD	5	5	5	5	5	5	5	5	5
		2000	5	0	0	NAD	5	5	5	5	5	5	5	5	5
MMC	I.P	1	5	0	0	NAD	–	–	–	–	–	5	5	5	5

*I.P*. intraperitoneal, *MMC* mitomycin C, *NAD* no abnormalities detected, *P.O*. per Os (oral gavage).

**Table 12:** Weight changes of animals in micronucleus test.

Test substance	Route	Dose (mg/kg)	Animal ID	Body weight(g) at the time	
				Group assignment	Sacrifice
Water for Injection	P.O	0	1101	34.58	35.01
			1102	36.36	36.68
			1103	36.59	36.65
			1104	37.97	38.05
			1105	38.01	38.21
			Mean	36.70	36.92
			S.D	1.41	1.30
MCT Oil	P.O	0	1201	35.22	35.63
			1202	36.32	36.85
			1203	36.59	36.61
			1204	37.73	37.45
			1205	38.02	37.41
			Mean	36.78	36.79
			S.D	1.13	0.74
MK-7	P.O	500	1301	35.24	34.45
			1302	36.19	37.39
			1303	36.60	37.61
			1304	37.54	37.91
			1305	38.15	38.48
			Mean	36.74	37.17
			S.D	1.14	1.57
	P.O	1000	1401	35.29	34.67
			1402	35.99	36.00
			1403	36.72	36.21
			1404	37.41	38.01
			1405	38.49	36.69
			Mean	36.78	36.32
			S.D	1.24	1.21
	P.O	2000	1501	35.34	36.19
			1502	35.92	36.48
			1503	36.86	35.90
			1504	37.20	36.97
			1505	38.55	38.40
			Mean	36.77	36.79
			S.D	1.24	0.98
MMC	I.P	1	1601	35.72	35.74
			1602	35.90	36.45
			1603	36.91	37.73
			1604	37.10	37.14
			1605	38.62	38.88
			Mean	36.85	37.19
			S.D	1.16	1.20

*I.P*. intraperitoneal, *MMC* mitomycin C, *P.O*. per Os (oral gavase), *S.D* standard deviation.

**Table 13:** Frequencies of micronucleated polychromatic erythrocytes and polychromatic erythrocytes in micronucleus test.

Test substance	Route	Dose (mg/kg)	Hour after administration	Animal ID	PCE	NCE	PCE/(PCE + NCE)	MNPCE/4000PCE
Water for injection	P.O	0	24	1101	256	244	0.512	0
				1102	250	250	0.500	1
				1103	234	266	0.468	1
				1104	228	272	0.456	3
				1105	265	235	0.530	0
				Mean	–	–	0.493	1
				S.D	–	–	0.031	1.22
MCT Oil	P.O	0	24	1201	247	253	0.494	0
				1202	280	220	0.560	0
				1203	260	240	0.520	0
				1204	247	253	0.494	2
				1205	247	253	0.494	0
				Mean	–	–	0.512	1
				S.D	–	–	0.029	0.60
MK-7	P.O	0	24	1301	216	284	0.432	0
				1302	250	250	0.500	30
				1303	248	252	0.496	0
				1304	260	240	0.520	0
				1305	278	222	0.556	1
				Mean	–		0.501	0.8
				S.D			0.045	1.30
	P.O	0	24	1401	249	251	0.498	1
				1402	284	216	0.568	1
				1403	275	225	0.550	4
				1404	248	252	0.496	1
				1405	293	207	0.586	0
				Mean	–	–	0.540	1.4
				S.D	–	–	0.041	1.52
	P.O	0	24	1501	243	257	0.486	0
				1502	223	277	0.446	1
				1503	248	252	0.496	2
				1504	238	262	0.476	1
				1505	265	214	0.553	0
				Mean	–	–	0.491	0.8
				S.D	–	–	0.047	0.84
MMC	I.P	0	24	1601	261	239	0.522	60
				1602	242	258	0.484	62
				1603	286	214	0.572	90
				1604	277	223	0.554	74
				1605	263	237	0.526	83
				Mean	–	–	0.532	73.8
				S.D	–	–	0.034	13.01

*I.P*. intraperitoneal, *MCT* medium chain triglyceride (vehicle control), *MMC* mitomycin C, *MNPCE* micronucleated polychromatic erythrocyte, *NCE* normochromatic erythrocyte, *PCE* polychromatic erythrocyte, *P.O*. per Os (oral gavase), *S.D.* standard deviation.

*Significantly different from negative control by Fisher's extract test (p < 0.05).

## Discussion

The origin of menaquinone-7 (MK7) is commonly found in fermented foods using microorganisms or animal-derived foods. MK7 is present in sources such as egg yolk, butter, milk, cow's liver, cheese, and natto, with natto being the richest source^5^. Natto is a traditional Japanese fermented soybean food made by fermenting cooked soybeans with Bacillus subtilis natto, and it is considered a archetypal dietary source of MK-7^6^. The intake of MK-7 in Western countries is likely to be less than 5 to 10% compared to the predominantly consumed vitamin K1 in foods. While all vitamin Ks have similar functions, there are differences in absorption potential and bioactivity^6^.

The intake of vitamin K1 and K2 is well known to play an important role in preventing the onset and progression of many diseases. Among them, vitamin K2 (MK-7) has been shown to have advantages over other vitamin K forms, considering its superior bioavailability and longer circulating half-life. The majority of available clinical studies still associate vitamin K1 with health impacts, while vitamin K2 has been shown to play a widespread role in tissues outside the liver, protecting against cardiovascular diseases, reducing the risk of cognitive disorders, and alleviating inflammation^6^.

The lack of adverse effects of MK-7 in healthy humans precluded the Institute of Medicine’s (IOM), the European Commission, the UK EVM, and the WHO/FAO from setting a Tolerable Upper Intake Level for any form of vitamin K, as levels of intake in excess of the Adequate Intake, even with additional intake of MK-7 from foods and supplements, cannot be interpreted as presenting a potential risk to health. The UK EVM recommended a Guidance Level of 1000 µg/d and the Council for Responsible Nutrition set a Tolerable Upper Intake Level for supplements of 10,000 µg/d^7^. As the use of MK-7 has increaed in recent years in nutritional and food supplements, high-dose toxicity studies are deemed necessary.

According to previous toxicity studies on MK-7, synthetic MK-7 exhibited low acute oral toxicity when orally administered to rats, with an LD50 of over 2000 mg/kg in mice, and no adverse effects were observed in subacute toxicity studies in Sprague–Dawley (SD) rats following administration of MK-7^3^. Furthermore, in acute, subacute, and genotoxicity studies conducted using Albino Wistar rats, toxicity was not observed in male and female rats administered MK-7 at a concentration of 2000mg/kg^2^.

In this study, the toxicity tests weres conducted to evaluate the safety of MK-7 (MediQ7, *Bacillus subtilis* var. *natto* culture extract-MK7) produced via microbial fermentation followed by a refining process. In the preliminary and main studies of the acute oral toxicity tests, all subjects survived at the dose of 5000 mg/kg with no toxic effects. Therefore, this dose was classified as "category 5" or "unclassified" in the GHS classification.

The 28-day repeated oral dose determination test was conducted to investigate the approximate toxicity observed after 28 days of repeated oral dosing and to utilize it as a basis for dose selection in the 90-day repeated dose toxicity study. The results showed no adverse effects attributable to MK-7 during the 28-day repeated oral dosing period. Based on this, it was deemed appropriate to set the high dose for the 90-day repeated dose toxicity study at 4500 mg/kg(the highest dose tested) or lower.

In the 90-day repeated dose toxicity study, toxicity potential was evaluated by assessing general observations, body weight, food consumption, hematological analysis, blood biochemical analysis, organ weights, gross necropsy findings, and histopathological changes. No statistically significant changes were observed in general observations, body weight, food consumption, and other parameters. This indicates that even with oral administration of MK-7, there was no interference with the metabolism of nutrients derived from food. In the blood biochemical tests, abnormal changes in total cholesterol, sodium, chlorine, triglycerides, and urea were observed in females and males of the main group, but there was no toxicological significance as the changes were within the range of biological fluctuations^8,9^. The thyroxine hormone level decreased in the male medium-dose group of the main group, which was considered an accidental occurrence as no dose-dependent changes were observed. On the basis of the results of organ weight measurement, the relative organ weight of the lungs in the recovery male high-dose group increased. However, because no abnormal changes were observed in the main group, there might be no toxicological significance. On the basis of findings from the autopsy, yellow spots on the left epididymis in the main male highdose group were observed in one case, which was confirmed as sperm granuloma by histopathological examination. The sperm granuloma observed in this test might be an accidental occurrence^10^.

To observe the reversibility, persistence, or delayed occurrence of toxic effects, a 28-day recovery period was conducted for both the control group and the 4500 mg/kg treatment group, with five males and five females included in each group. The increase in albumin and abnormal changes in the albumin/globulin ratio observed in the recovery group were considered to have no toxicological significance because no abnormal changes were observed in the main group. The relative organ weight of the lungs increased in the high-dose male recovery group, but no abnormal changes were observed in the main group, indicating no toxicological significance. On the basis of the results of hematological tests, reticulocytes were reduced in the recovery male vehicle control group and prothrombin time was increased in the recovery female high-dose group. However, because abnormal changes were observed in the main group, there no toxicological significance. These results indicate that MK-7 is reversible and does not exhibit delayed toxicity.

In the bacterial reverse mutation test, the test substance, did not induce reverse mutation in the bacterial strains under the main test conditions. In the main study of the chromosomal aberration assay, the number of chromosomally abnormal cells did not increase significantly in the test substance treatment groups with or without a metabolic activation system compared to the negative control group. In the micronucleus test, the test substance did not induce micronuclei in the bone marrow cells of mice under the main test conditions.

In conclusion, in this study, which was conducted at an institution operating in accordance with the OECD Principles of Good Laboratory Practice, various toxicological evaluations confirmed the lack of genotoxicity of MK-7 and determined its NOAEL to be 4500 mg/kg bw/day.

## Methods

### Materials

The test item was MK-7 extracted from *Bacillus subtilis* var. *natto* culture (MediQ7, GF Fermentech, South Korea). It is produced through fermention by *Bacillus subtilis* var. *natto* and subsequent purification. The purity of MK-7 utilized in this study was over 96%. The MK-7 was added to medium chain triglyceride (MCT) oil (Musim Mas) at the concentration of 50,000 mg/kg and was kept in low temperature, light-shielding storage conditions. (Table 14) MCT oil was considered an excipient in the toxicity study. All other chemicals were commercially available with confirmed purity levels.

**Table 14:** General descriptive characteristics of *Bacillus subtilis natto* culture extract-MK7(MK-7).

Parameter	Description*
Source	*Bacillus subtilis* var. *natto*
Synonyms	Vitamin K2-MK7; vitamin K2; menaquinone-7 (USP grade); MK-7
Trade name	MediQ7
Systematic name	(all-E)-2-(3,7,11,15,19,23,27-heptamethyl-2,6,10,14,18,22,26-octacosaheptaenyl)-3-methyl-1,4-naphthalenedione
CAS No	2124-57-4
Chemical formula	C_46_H_64_O_2_
Molecular weight	649 g/mol
Content (menaquinone-7)	53,157 mg/kg
Appearance	Yellowish oil
Color	Yellowish
Odor	Characteristic
Taste	Characteristic
Storage	In cool temperature in a dry and dark place, and away from high heat, humidity, and light
Carrier	Medium chain triglyceride (MCT) oil

*Based on information provided GF Fermentech.

#### Preparation and analysis of the dosing formulations

The test article was weighed on an electronic balance (BCE2202I-1SKR, Sartorius, Germany). Afterwards, the MCT oil was added to prepare the article into the stipulated concentrations. The homogeneity of the prepared articles and stability (room temperature: 7 days) of the low (4 mg/mL) and high dose (920 mg/mL) formulations were assured through a validation of the analytical methods and stability test for the concentration of the prepared test items (Study No.: C19ZV-387N). Considering its assured storage conditions (temperature : 15–25 °C), the test article were prepared at least once a week. The concentration analyzes were measured by taking three samples in the middle layer for each dosing formulation before dosing and at week 13 dosing. The precision of the quantitative value for the dosing formulation was 0.45–2.34% (Prior to dosing: 0.45–1.27%, week 13: 1.17–2.34%) and the accuracy was 85.7–113.0% (Prior to dosing: 0.45–1.27%, week 13), which satisfied the validation study’s criterion.

### Animals

The oral toxicity tests utilizing rodents were conducted with Sprague–Dawley (SD) rats(Rattus norvegicus), following the guidelines outlined in "OECD Guideline for Testing of Chemicals No.420, Acute Oral Toxicity-Fixed Dose Procedure" (adopted: December 17, 2001) and “OECD Guideline For The Testing Of Chemicals No. 408, Repeated Dose 90-Day Oral Toxicity Study in Rodents” (adopted: Jun. 25, 2018). And the micronucleus test were conducted with ICR mice, following the guidelines outlined in "OECD Guideline for Testing of Chemicals No.474, Mammalian Erythrocyte Micronucleus Test" (adopted: Jul. 29, 2016). Additionally, all studies were performed in compliance with the OECD Principles of Good Laboratory Practice (ENV/MC/CHEM (98)17 as revised in 1997). The Globally Harmonized System of Classification and Labelling of Chemicals (GHS) categories was utilized to assess MK-7’s hazardous properties in the context of its OECD 420 results. All animal was approved by the Animal Testing and Operation Ethics Committee in accordance with the Animal Protection Act (No. 16977 Revised on Feb. 11, 2020). The contents of this manuscript fulfills the reporting recommendations of the ARRIVE guidelines.

SD rats (Specific pathogen-free grade, male and female, 5–6 week-old) were purchased from ORIENTBIO INC., Korea (322, Galmachi-ro, Jungwon-gu, Seongnam-si, Gyeonggi-do, Korea). Upon the acquisition of animals, the number of animals was confirmed, and the body weight was measured after the examination for any abnormal symptoms. Materials such as test reports provided by the animal supplier were preserved as a raw data for the experiment. All animals were quarantined and acclimatized for 3 days and were then moved to the animal room and allowed to acclimatize for 5 or more days (male: 5 days, female: 6 days). During the acclimation period, the clinical signs of all animals were observed once a day. The animals were uniquely identified by an indelible marking on the tail (in quarantine and acclimation: red, after group assignment: black or blue) Cages were individually identified by ID cards.

Only animals without abnormalities in the comparison of weights at receipt and group assignment, and without abnormal symptoms during the period of acclimation were used for this study. During acclimation period, the body weight of healthy animals was measured and animals exceeding ± 20% of the group’s mean weight were excluded. The selected animals were grouped and separated in a zigzag pattern based on their ranked weights to ensure a as even as possible distribution of group weights.

A total of 185 rats (5 in Acute Oral toxicity test, 50 in the Repeat dose 28-day oral dose determination test, and 130 in the Repeat dose 90-day oral toxicity test) were used. During the studies, animals were housed in wire cages (width 19.5 cm × length 43 cm × height 20 cm), with 1 rat per cage. Temperature (22 ± 3 ℃), relative humidity (30–70%), air cycling(10–15 times/hr), illumination time (12 h), and illumination (150–300 lx) were maintained, and sterilized solid rat food(2918C, Envigo, USA) was freely fed. In addition, water was put into a water supply bottle made of polycarbonate and was freely consumed.

### Toxicological test methods

#### Acute oral toxicity test

The acute oral toxicity test was performed according to “OECD Guideline for Testing of Chemicals No.420, Acute Oral Toxicity-Fixed Dose Procedure" (adopted: December 17, 2001). According to a previous toxicity studies, the sublethal dose (LD50) of MK-7 is known to exceed 2000 mg/kg^2,3^. Therefore, the starting dose for the preliminary test was set at 5000 mg/kg. For the acute toxicity study, a total of five 6-week-old female SD rats were used (one for the preliminary test and four for the main test). The dose was calculated based on the body weight after fasting approximately 12 h or more before administration of the test substance (the day of administration). At this time, about 5.26 mL/kg was forcibly administered into the stomach once with a disposable syringe fitted with intubation tubes in consideration of the specific gravity (0.95 g/mL) of the test substance. The preliminary test involved one rat, after confirming survival for 3 days, a main study was conducted in which four animals were administered the same dose. Observations, including general condition (signs of toxicity, onset of symptoms, recovery period), body weight changes, and mortality, were recorded for all animals on the day of administration (day 0) and at 30 min, 1, 2, 3, and 4 h post-dosing. After 14 days of observation, the surviving five animals were asphixiated with CO_2_ gas, euthanized through bleeding from the posterior vena cava/abdominal aorta, autopsied, and visually examined all organs and tissues. Statistical analysis was not performed.

#### Repeated dose oral toxicity tests

##### 28-day, repeated (oral) dose determination test

In this test, 25 male and 25 female SD rats, each 6 weeks old, were utilized. At the start of dosing, the male weight ranged from 217.25 g to 246.62 g, and the female weight was between 156.47 and 173.93 g.

Overall toxicity of repeated dose administration of MK-7 over 28 day in SD rats was investigated to provide a basis for setting the dose to be used in the repeated dose 90-day oral toxicity test. In a single oral dose toxicity study, when 5000 mg/kg of the test article was administered to the test system, temporary diarrhea was observed, but no other abnormal changes and deaths were observed. Thus, in this study, the high dose group was determined at 4500 mg/kg and ratio of 3 was applied to set the mid dose group at 1500 mg/kg and the low dose group at 500 mg/kg. Also, a vehicle control group and a negative control group were additionally set to confirm any changes due to the vehicle. The administration method involved oral (gavage) administration into the stomach using disposable syringes fitted with intubation tubes. The test substance was administered once a day, seven times a week, for a duration of 28 days. During the observation period, general symptoms, body weight, and feed consumption were observed. After the observation period, hematological examination, blood biochemical examination, organ weight measurement, and visual inspection during necropsy were performed. At the end 28 days, all surviving animals were fasted more than 12 h(overnight) prior to euthansia. The animals were anesthetized with isoflurane(Ifran solution, HANA PHARM Co., LTD., Lot No.: 0019) and blood samples were collected from the abdominal aorta. For clinical pathological examination, the abdominal aorta and inferior vena cava of the rats, from which blood was drawn, were cut to exsanguinate and kill them. All organs and tissues(thymus, liver, teste, ovary, epididymis, adrenal gland, spleen, kidney, heart, uterus, lung) of the exterior, head, thoracic cage, and abdominal cavity were grossly observed.

##### 90-day, repeated dose (oral) toxicological test with 28-day recovery group

This study aimeds to investigate the toxic effects of repeated dose 90-d oral (gavage) exposure of SD rats to MK7, on target organs and to determine the no-observed-adverse-effect level (NOAEL) according to the OECD Guideline For The Testing Of Chemicals No. 408, Repeated Dose 90-Day Oral Toxicity Study in Rodents” (Adopted: Jun. 25, 2018). In this test, 65 male and 65 female SD rats, each 6 weeks old, were used. At the start of dosing, the male weight ranged from 180.63 to 215.83 g, and the female weight was between 134.09 and 164.57 g. The test substance was administered orally (gavage) once a day, seven times a week, for 90 days. The dose was calculated based on the most recently measured body weight. The test groups were configured as low-dose (500 mg/kg), a medium-dose (1500 mg/kg), high-dose (4500 mg/kg) groups, avehicle(excipient) control group, and a negative control group, (sterile water administered via injection). In the main group, each test group included 10 males and 10 females. An additional recovery group for each control and the high-dose group (5animals/sex/group) was included. The administration method involved forced oral administration into the stomach using disposable syringes fitted with intubation tubes. The test substance was administered once a day, seven times a week, for a duration of 91 days.

During the study period, general symptoms were observed twice a day (morning and afternoon) for all animals. Body weight measurements were taken using an electronic balance (BCE2202-1SKR, Sartorius, Germany) for all animals on the initiation day of administration (pre-dose), once a week after initiation, on the day before necropsy (pre-fasting), and on the day of necropsy (post-fasting). Body weight data on the day of necropsy were not included in the evaluations of body weights since these data were fasted body weights of all animals. Food and water consumption was recorded using an electronic balance (BCE2202-1SKR, Sartorius, Germany) once a week during the study period from the start of administration. The amounts of food/water supplied on the day and the amounts of food/water remained on the following day were measured, and then the difference between the two amounts was calculated as food/water consumption. Functional observation battery was performed on all surviving animals after 11 weeks of administration. Ophthalmological examinations were conducted in the negative control group, vehicle control group and high-dose groups, prior to dosing, during the last week of administration, and during the last week of the recovery period. inducing pupillary dilation by dropping a mydriatic agent (Mydriacyl Eye Drops 1%: Lot No.: 19B12UA) into both eyeballs, the anterior segment, the transparent media, and the fundus of the eye were observed using an ophthalmoscope (GENESIS-D, KOWA, Japan). Urine analysis was conducted on five males and five females from each group, selected consecutively starting from the first animal number, during the last week of administration and the last week of the recovery period. Fresh urine (urine within approximately 3 h of excretion) and overnight urine (urine within approximately 24 h of excretion) were collected for analysis. Animals were fasted during collection of fresh urine samples but had free access to water. Urinalysis parameters (urine volume, glucose, bilirubin, nitrite, protein, pH, specific gravity, color, urobilinogen, occult blood, ketones, clarity, urinary sediment, and leukocyte) were evaluated using a urine chemistry analyzer(UC-1000, Sysmex, Japane). An amount of urines was measured using a syringe and sediment test was performed using a microscope.

All surviving animals were fasted more than 12 h(overnight) prior to necropsy. Prior to the collecting blood samples, the animals were anesthetized with isoflurane(Ifran solution, HANA PHARM Co., LTD., Lot No.: 0019) and blood samples were collected from the abdominal aorta. For clinical pathological and histopathological examinations, the abdominal aorta and inferior vena cava of the rats, from which blood was drawn, were cut to phlebotomize and kill them. All organs and tissues(thyroids gland, thymus, liver, testes, ovaries, brain,pituitary gland, epididymides, adrenal glands, spleen, kidneys, heart, uterus, prostate, seminal vesicle, lung) of the exterior, head, thoracic cage, and abdominal cavity were assessed in a gross necropsy examination.

#### Genotoxicity tests

##### Bacterial reverse mutation test

A bacterial reverse mutation test was performed using a histidine-requiring Salmonella typhimurium strains typhimurium (TA98, TA100, TA1535, TA1537) and a tryptophan-requiring *Escherichia coli* (WP2uvrA/pKM101) strain to determine the genotoxicity of MK-7. The study was performed according to OECD Guideline For Testing Of Chemicals No. 471, Bacterial Reverse Mutation Test (Adopted: Jul. 21, 1997, Corrected: Jun. 26, 2020). All strains in the presence and absence of a metabolic activation system were set at five levels of dose progression, with a maximum dose of 5000 µg/plate in the main study. Positive substances were selected according to the recommendations in OECD 471.

The strain used in this test was purchased from Molecular Toxicology Inc. and was subcultured by Croen after confirming the characteristics. Each strain was inoculated into nutrient broth liquid medium and cultured using a shaking incubator (BF-60SIR, Biofree, Korea). After the cultivation, the bacterial culture and dimethyl sulfoxide (DMSO) were mixed at a ratio of approximately 9%, aliquoted into freezing tubes, and stored in a liquid nitrogen tank. One frozen strain was selected to confirm the genotype of each strain. Strains with confirmed characteristics were used in the experiment. Hepatic homogenates, isolated from the liver of male SD rats, were utilized for metabolic activity were purchased from Molecular Toxicology, Inc. The positive control substances used were sodium azide (SA), 2-nitrofluorene (2-NF), 2-aminoanthracene (2-AA), benzo[a]pyrene (B[a]P), 9-aminoacridine (9-AA), and 2-(2-furyl)-3-(5-nitro-2-furyl) acrylamide (AF-2), following the guidelines. In the dose-setting preliminary test, the test substance was set at the maximum dose of 5000 μg/plate, and a total of 7 doses were set, including the maximum dose, in a ratio of 4 (1.22 μg/plate, 4.88 μg/plate, 19.5 μg/plate, 78.1 μg/plate, 312.5 μg/plate, 1250 μg/plate, and 5000 μg/plate). No precipitation or growth inhibition by the test substance was observed at any dose for all strains with or without the presence of metabolic activation. Therefore, the dose setting for the main test for all strains with or without metabolic activation involved setting the maximum dose at 5000 μg/plate, with a total of 5 doses in a twofold ratio (312.5 μg/plate, 625 μg/plate, 1250 μg/plate, 2500 μg/plate, 5000 μg/plate). This included negative and positive control groups, with the negative control group using tetrahydrofuran (THF) as a vehicle, and three plates being used for each dose. The frozen strains were thawed and inoculated into 2.5% nutrient broth liquid medium, then cultured in a shaking incubator at approximately 37 °C. After culturing, the absorbance of each strain was measured using an ELISA reader (wavelength: 600 nm, EPOCH, Biotek, U.S.A.). Additionally, strains with a viable cell count of 1 × 10^9^ cells/mL or higher were used.

In the presence of a metabolic activation system, test substances and negative control substances were added to test tubes at 0.02 mL each, followed by the addition of sterile distilled water at 0.08 mL. Positive control substances were added at 0.1 mL each. To each test tube, 0.5 mL of S9 mix and 0.1 mL of bacterial culture were added. For *Salmonella* strains, 2 mL each of *Salmonella* top agar was added, and for *E. coli*, 2 mL each of *E. coli* top agar was added, followed by vortexing. The mixture was then evenly poured onto minimal glucose agar plates and allowed to solidify. In the absence of a metabolic activation system, 0.2 M sodium phosphate buffer (pH 7.4) was added instead of S9 mix at 0.5 mL. The subsequent treatment followed the same procedure as in the presence of a metabolic activation system. After solidification of the top agar, the plates were inverted and incubated at approximately 37 °C for about 48 h.

The criteria for determining growth inhibition was defined as a significant decrease in colony count compared to the negative control group, a noticeable reduction in the size of clusters, or a substantial decrease in the background lawn when compared to the negative control group. Tests were judged as positive if the colony count of revertant colonies was more than twice that of the negative control group or showed dose-dependent or reproducible increases in at least one strain and at one dose, whether or not a metabolic actication was present.

The number of revertant colonies was recorded, and the mean and standard deviation were calculated. No statistical analysis was performed.

##### Chromosomal aberration test

The main study was conducted by exposing Chinese hamster lung cells (a mammalian cell line) to the test substance and observing the chromosomal structure and numerical aberrations to evaluate genotoxicity, according to the OECD Guideline For The Testing Of Chemicals No. 473, In vitro Mammalian Chromosomal Aberration Test(Adopted: Jul. 29, 2016). To determine the maximum dose for the main study, a preliminary study was conducted with seven levels at a dose progression factor of 2 (31.3, 62.5, 125, 250, 500, 1000, and 2000 μg/mL). In the preliminary study, no growth inhibition was observed in the test substance treatment groups with or without a metabolic activation system. However, precipitation of the test substance was observed at 250 to 2000 μg/mL in the short-term treatment group with or without a metabolic activation system, and 125 to 2000 μg/mL in the continuous treatment group without a metabolic activation system. Therefore, 250 μg/mL in the short-term treatment group with or without a metabolic activation system and 125 μg/mL in the continuous treatment group without a metabolic activation system were set as the maximum doses for the main study. As a positive control, mitomycin C (MMC) was prepared with sterile water for injection (WFI), and Benzo[a]Pyrene was prepared using DMSO. The prepared positive control substances were aliquoted into brown tubes, frozen, stored at − 80 to − 60 °C in a deep freezer (DW86L-338, Haier, China), and thawed on the day of treatment. The liver of male rats induced with Aroclor 1254 (Monsanto KL615) was used for metabolic activation system (S9). Cells treated with the negative control substance, excipient, test substance, and positive control substances were isolated and centrifuged at 1000 rpm for 5 min. Test specimens were prepared using isolated cells. Two plates were used per dose.

In the main study, cell with fewer than 24 passages were used for the chromosomal aberration test, and the colcemid (final dose of 0.2 μg/mL) solution was treated 2 h before the end of culture. The cultured cells were washed with phosphate buffered saline and treated with a 0.05% trypsin- ethylenediaminetetraacetic acic (EDTA) solution to separate the cells from the bottom of the plate. The separated cells were centrifuged at 1000 rpm for 5 min. After discarding the supernatant, 3 mL of a 0.075 mol/L KCl solution, kept at 37 °C, was added, followed by treatment at 37 °C for 30 min. Next, 1 mL of a cooled fixative (methanol:acetic acid ratio of 3:1) was added to the solution, which was centrifuged at 1000 rpm for 5 min, and the supernatant was removed to prefix the cells. Thereafter, 5 mL of a cooled fixative was added and centrifuged at 2000 rpm for 5 min. This process was repeated once more to fix the cells. A specimen slide was prepared by dropping the cell suspension onto a glass slide. Two slide specimens were prepared for each plate. After drying, the slide was stained with a 5% Giemsa staining solution for 20 min, washed with distilled water, and dried. One of the specimen slides was coded and inspected. Structural aberration and numerical aberration were observed under a microscope (Eclipse E200, Nikon, Japan) at 1000 × magnification for 150 metaphase cells per coded specimen slide. The gap was classified and counted as a deletion when the gap was narrower than the width of the chromosome but it was not included in the structural aberration and comprehensive evaluation when recording the result of chromosomal aberration.

##### In vivo mouse micronucleus test

The main study was conducted to examine chromosomal aberration or mitotic aberration induced by the test substance in vivo on the micronucleus of Institute of Cancer Research (ICR) mouse bone marrow cells according to the “OECD Guideline For the Testing of Chemicals, No. 474 Mammalian Erythrocyte Micronucleus Test” (Adopted: July 29, 2016) to further assess the genotoxicity of MK-7. To determine the maximum dose in the main study, a preliminary study was conducted using the doses of 500, 1000, and 2000 mg/kg for males and females. As a result, no general symptoms or deaths were observed in treated animals. Therefore, in the main study, only male animals were used, with 2000 mg/kg being the highest dose of the three levels at a dose progression factor of 2 (500, 1000, and 2000 mg/kg). A negative control group, an vehicle control group, and a positive control group (MMC) were additionally utilized. The positive control substance was dissolved in sterile water for injection at an administration dose of 1 mg/kg. All animals were observed for general symptoms and death rates at least twice a day during the administration period. After the last administration, general symptoms were observed for one day, and morbidity and mortality were monitored. Body weight was measured before administration and before bone marrow cell collection. A test substance was considered positive if all of the following three criteria were met, including:.

1. The proportion of polychromatic erythrocytes with micronuclei increased compared to the negative control group, which was administered at least one dose in the test substance group at the same time.
2. The proportion of polychromatic erythrocytes with micronuclei increased in a dose-dependent manner according to the dose in the test substance group.
3. At least one of the results showed a distribution in which the proportion of polychromatic erythrocytes with micronuclei deviated from the historical control data of the negative control group.

### Statistical analysis

Parametric multiple comparison procedures were subjected to one-way ANOVA test for significance, which was judged at a probability value of p < 0.05. Ifthe test showe significant difference, it was subjected to the Levene’s test for equality of variances. If homoscedasticity of the data was accepted, Scheffe multiple range test was applied. In cases of significant deviation of variance homogeneity, Dunnett’s T3-test was applied for post-hoc analysis. All statistical analyses were performed with widely used commercial statistical package, SPSS software (IBM^®^ SPSS Statistics, ver. 24).

For the aberration cell data to chromosomal aberration test, a Fisher’s exact test was used for comparison of the vehicle group with the substance group, negative control group with the test substance group or the vehicle group with the positive control group. Positive result was evaluated according to increase of dose–dependent and pvalue by statistical analysis. The statistical significance was not the only determining factor for a positive response and biological relevance of the results was considered first.

The frequency of induction of micronuclei was verified using the Fisher's exact test (significance level of 0.05). Appearance frequency and weight of polychromatic erythrocytes were determined by performing ANOVA after the normality test (significance level of 0.05).

### Supplementary Information

Supplementary Table S1.

## Data Availability

The datasets supporting the conclusions of this article are included within the article.
