# Supplementary material for: Safety evaluation of vitamin K2 (menaquinone-7) via toxicological tests
Source: Sci Rep. 2024 Mar 5;14:5440. doi: 10.1038/s41598-024-56151-w (PMC10914784; doi:10.1038/s41598-024-56151-w)
Supplement: Supplementary file 1 — Supplementary Table S1. [file 41598_2024_56151_MOESM1_ESM.docx]

Table S1. Chromosomal aberration: structural arrangement test results in the main study

| Test Article | S9 mix | Dose (ug/mL) | No. of Cell analyzed | ctg | ctb | cte | csg | csb | | cse | Frg/  other | Gap(-) aberration cell | | Gap(+) aberration cell | | Total Normal cell |
| --- | --- | --- | --- | --- | --- | --- | --- | --- | --- | --- | --- | --- | --- | --- | --- | --- |
|  |  |  |  |  |  |  |  |  |  |  |  | No. | % | No. | % |  |
| Water for Injection | + 6h | 0 | 150 | 0 | 0 | 0 | 0 | 0 | | 0 | 0 | 0 | 0.0 | 0 | 0.0 | 300 |
|  |  |  | 150 | 0 | 0 | 0 | 0 | 0 | | 0 | 0 | 0 |  | 0 |  |  |
| Acetone |  | 0 | 150 | 0 | 0 | 0 | 0 | 0 | | 0 | 0 | 0 | 0.0 | 0 | 0.0 | 300 |
|  |  |  | 150 | 0 | 0 | 0 | 0 | 0 | | 0 | 0 | 0 |  | 0 |  |  |
| MK-7 |  | 62.5 | 150 | 1 | 0 | 0 | 0 | 0 | | 0 | 0 | 0 | 0.3 | 1 | 0.7 | 298 |
|  |  |  | 150 | 0 | 1 | 0 | 0 | 0 | | 0 | 0 | 1 |  | 1 |  |  |
|  |  | 125 | 150 | 1 | 0 | 0 | 0 | 0 | | 0 | 0 | 0 | 0.0 | 1 | 0.3 | 299 |
|  |  |  | 150 | 0 | 0 | 0 | 0 | 0 | | 0 | 0 | 0 |  | 0 |  |  |
|  |  | 250 | 150 | 0 | 0 | 1 | 0 | 0 | | 0 | 0 | 1 | 0.3 | 1 | 1.0 | 297 |
|  |  |  | 150 | 2 | 0 | 0 | 0 | 0 | | 0 | 0 | 0 |  | 2 |  |  |
| B[a]P |  | 20 | 150 | 10 | 2 | 23 | 1 | 1 | | 0 | 0 | 24 | 14.3^*^ | 31 | 19.3 | 242 |
|  |  |  | 150 | 8 | 1 | 18 | 2 | 0 | | 1 | 0 | 19 |  | 27 |  |  |
| Water for Injection | - 6h | 0 | 150 | 0 | 0 | 0 | 0 | 0 | | 0 | 0 | 0 | 0.0 | 0 | 0.7 | 298 |
|  |  |  | 150 | 2 | 0 | 0 | 0 | 0 | | 0 | 0 | 0 |  | 2 |  |  |
| Acetone |  | 0 | 150 | 2 | 0 | 0 | 0 | 0 | | 0 | 0 | 0 | 0.0 | 1 | 0.3 | 299 |
|  |  |  | 150 | 0 | 0 | 0 | 0 | 0 | | 0 | 0 | 0 |  | 0 |  |  |
| MK-7 |  | 62.5 | 150 | 0 | 0 | 0 | 0 | 0 | | 0 | 0 | 0 | 0.0 | 0 | 0.0 | 300 |
|  |  |  | 150 | 0 | 0 | 0 | 0 | 0 | | 0 | 0 | 0 |  | 0 |  |  |
|  |  | 125 | 150 | 0 | 0 | 0 | 0 | 0 | | 0 | 0 | 0 | 0.0 | 0 | 0.0 | 300 |
|  |  |  | 150 | 0 | 0 | 0 | 0 | 0 | | 0 | 0 | 0 |  | 0 |  |  |
|  |  | 250 | 150 | 0 | 0 | 0 | 0 | 0 | | 0 | 0 | 0 | 0.3 | 0 | 0.7 | 298 |
|  |  |  | 150 | 1 | 1 | 0 | 0 | 0 | | 0 | 0 | 1 |  | 2 |  |  |
| MMC |  | 0.1 | 150 | 10 | 0 | 29 | 0 | 1 | | 0 | 0 | 28 | 20.7^*^ | 33 | 25.0 | 225 |
|  |  |  | 150 | 15 | 2 | 35 | 1 | 0 | | 1 | 0 | 34 |  | 42 |  |  |
| Water for Injection | - 24h | 0 | 150 | 0 | 0 | 0 | 0 | 0 | | 0 | 0 | 0 | 0.0 | 0 | 0.0 | 300 |
|  |  |  | 150 | 0 | 0 | 0 | 0 | 0 | | 0 | 0 | 0 |  | 0 |  |  |
| Acetone |  | 0 | 150 | 0 | 0 | 0 | 0 | 0 | | 0 | 0 | 0 | 0.0 | 0 | 0.0 | 300 |
|  |  |  | 150 | 0 | 0 | 0 | 0 | 0 | | 0 | 0 | 0 |  | 0 |  |  |
| MK-7 |  | 31.3 | 150 | 0 | 0 | 0 | 0 | 0 | | 0 | 0 | 0 | 0.0 | 0 | 0.0 | 300 |
|  |  |  | 150 | 0 | 0 | 0 | 0 | 0 | | 0 | 0 | 0 |  | 0 |  |  |
|  |  | 62.5 | 150 | 0 | 0 | 0 | 0 | 0 | | 0 | 0 | 0 | 0.0 | 0 | 0.3 | 299 |
|  |  |  | 150 | 1 | 0 | 0 | 0 | 0 | | 0 | 0 | 0 |  | 1 |  |  |
|  |  | 125 | 150 | 3 | 0 | 0 | 0 | 0 | | 0 | 0 | 0 | 0.0 | 2 | 1.3 | 296 |
|  |  |  | 150 | 2 | 0 | 0 | 0 | 0 | | 0 | 0 | 0 |  | 2 |  |  |
| B[a]P |  | 0.1 | 150 | 20 | 7 | 35 | 2 | 0 | | 1 | 0 | 39 | 23.7^*^ | 52 | 32.0 | 204 |
|  |  |  | 150 | 18 | 3 | 32 | 1 | 0 | | 0 | 0 | 32 |  | 44 |  |  |
| B[a}P: Benzo[a]pyrene, MMC: Mitomycin C | | | | | | | | |  | | | | | | | |
| *: p<0.05, significant differences between control and treatment group by Fisher’s exact test | | | | | | | | | | | | | | | | |
